# The Apparent Diffusion Coefficient (ADC) is a useful biomarker in predicting metastatic colon cancer using the ADC-value of the primary tumor

**DOI:** 10.1371/journal.pone.0211830

**Published:** 2019-02-05

**Authors:** Elias Nerad, Andrea Delli Pizzi, Doenja M. J. Lambregts, Monique Maas, Sharan Wadhwani, Frans C. H. Bakers, Harrie C. M. van den Bosch, Regina G. H. Beets-Tan, Max J. Lahaye

**Affiliations:** 1 University of Maastricht and GROW School of Oncology and Developmental Biology, Maastricht, The Netherlands; 2 Department of Radiology, The Netherlands Cancer Institute, Amsterdam, The Netherlands; 3 Department of Radiology, Addenbrookes Hospital Cambridge University Hospitals NHS trust, Cambridge, United Kingdom; 4 Institute for Advanced Biomedical Technology (ITAB), Gabriele d'Annunzio University, Chieti, Italy; 5 Department of Radiology, The Netherlands Cancer Institute, Amsterdam, The Netherlands; 6 Department of radiology, Queen Elizabeth Hospital, University Birmingham Hospitals NHS trust, Birmingham, United Kingdom; 7 Department of Radiology, Maastricht University Medical Centre, Maastricht, The Netherlands; 8 Department of Radiology, Catharina Hospital, Eindhoven, The Netherlands; Karmanos Cancer Institute, UNITED STATES

## Abstract

**Purpose:**

To investigate the role of the apparent diffusion coefficient (ADC) as a potential imaging biomarker to predict metastasis (lymph node metastasis and distant metastasis) in colon cancer based on the ADC-value of the primary tumor.

**Methods:**

Thirty patients (21M, 9F) were included retrospectively. All patients received a 1.5T MRI of the colon including T2 and DWI sequences. ADC maps were calculated for each patient. An expert reader manually delineated all colon tumors to measure mean ADC and histogram metrics (mean, min, max, median, standard deviation (SD), skewness, kurtosis, 5th-95th percentiles) were calculated. Advanced colon cancer was defined as lymph node mestastasis (N+) or distant metastasis (M+). The student Mann Whitney U-test was used to assess the differences between the ADC means of early and advanced colon cancer. To compare the accuracy of lymph node metastasis (N+) prediction based on morpholigical criteria versus ADC-value of the primary tumor, two blinded readers, determined the lymph node metastasis (N0 vs N+) based on morphological criteria. The sensitivity and specificity in predicting lymph node metastasis was calculated for both readers and for the ADC-value of the primary tumor, with histopathology results as the gold standard.

**Results:**

There was a significant difference between the mean ADC-value of advanced versus early tumors (p = 0.002). The optimal cut off value was 1179 * 10^−3^ mm2/s with an area under the curve (AUC) of 0.83 and a sensitivity and specificity of 81% and 86% respectively to predict advanced tumors. Histogram analyses did not add any significant additional value.

The sensitivity and specificity for the prediction of lymph node metastasis based on morphological criteria were 40% and 63% for reader 1 and 30% and 88% for reader 2 respectively. The primary tumor ADC-value using 1.179 * 10^−3^ mm2/s as threshold had a 100% sensitivity and specificity in predicting lymph node metastasis.

**Conclusion:**

The ADC-value of the primary tumor has the potential to predict advanced colon cancer, defined as lymph node metastasis or distant metastasis, with lower ADC values significantly associated with advanced tumors. Furthermore the ADC-value of the primary tumor increases the prediction accuracy of lymph node metastasis compared with morphological criteria.

## Introduction

Surgical resection of the primary tumor and regional lymph nodes is the cornerstone of curative treatment for colon cancer. After surgical resection, adjuvant chemotherapy is considered in patients with stage II or III disease to reduce the risk of disease recurrence (i.e. patients with locally advanced tumors and/or lymph node metastasis). In rectal cancer, *neo*adjuvant (i.e. preoperative) instead of adjuvant (i.e. postoperative) chemoradiotherapy has become the standard of care in the United States and Europe after the German rectal cancer trial, which showed improved local control and reduced toxicity with *neo*adjuvant chemoradiotherapy, compared to adjuvant chemoradiotherapy [1]. Similar results were found in advanced colon cancer patients in several smaller studies [2–6]. Therefore a large multicenter study called FOXTROT-trial [7] is currently investigating whether these promising results of neoadjuvant therapy can improve the outcome for advanced colon cancer patients similarly as with rectal cancer patients. If so, preoperative imaging will play an important role in the selection of advanced colon cancer patients for neoadjuvant treatment. Currently, staging of colon cancer is performed with computed tomography (CT) however, a recent meta-analysis showed unsatisfactory results for CT in identifying high-risk factors such as T- and especially N-staging [8]. MRI might be a logical alternative to CT because of its superior soft tissue contrast and the fact that MRI is already well known as a reliable staging modality to identify advanced tumors in rectal cancer [9]. To date, only a small number of studies investigated MR imaging for the staging of colon cancer patients, with promising results considering T-staging. Unfortunately accurate N-staging (i.e prediction of lymph node metastasis) remains a problem [10–12]. It needs to be addressed that these studies focused solely on morphological changes for T and N staging (i.e. visible breach of the muscularis propria layer of the bowel wall for T3 tumors, short axis diameter/shape of lymph nodes for nodal staging). However predicting lymph node metastasis with this standard approach is thwarted by microscopic metastasis which increase false negative results and conversely enlarged benign (inflammatory) lymph nodes which increase false positive results. Functional MR imaging by obtaining additional quantitative imaging ‘biomarkers’, such as the Apparent Diffusion Coefficient (ADC) may aid radiologists in their diagnosis. The ADC value is a quantitative measure of the magnitude of movement of water protons within a given tissue[13]. This movement due to diffusion is influenced mainly by tissue cellularity, integrity of cell membranes and viscosity of fluids, thereby providing an indirect measure of a tissue’s microcellular architecture. ADC measurements are reliably demonstrated to be a beneficial predictor of tumor aggression in rectal cancer[14–17] and other types of cancer such as breast [18–20] and prostate cancer[21–23]. Therefore, our study investigates the value of ADC as a biomarker to identify advanced colon cancer, defined as tumors with metastatic potential (lymph node metastasis and distant metastasis) and whether it can provide additional value in predicting lymph node metastasis compared with morphological staging only. Due to the heterogeneity of cancer tumors however, mean and median ADC values might not be representative. That is why our secondary aim is to evaluate if adding histogram ADC analyses as a post processing step could be of added value.

## Methods and materials

Due to the retrospective nature of the study informed consent was waived and the study was approved by the Maastricht University Medical Center Medical Review Ethics Committee.

### Patients

From April 2014 until May 2015 consecutive patients diagnosed with colon cancer at Maastricht University Medical Center were considered for inclusion in this study. A total of thirty patients, 21 male and 9 female with an average age of 71 years (range 54–83 years) were retrospectively included in the final analysis (Table 1) based on the following inclusion criteria: (a) biopsy-proven adenocarcinoma of the colon (with a distal tumor margin > 15 cm from the anorectal junction, measured at endoscopy), (b) preoperative staging with a standardized MRI protocol including DWI and ADC sequences, (c) surgical resection of the tumor or signs of metastatic disease on staging imaging or during surgery.

**Table 1:** Baseline patient characteristics.

**Gender**	
**Female**	9
**Male**	21
**total**	30
**Age, years**	
**Range**	54–83
**Mean**	71
**Median**	70
**Tumor Location**	
**Ceacum**	6
**Ascending colon**	4
**Transversum colon**	4
**Descending colon**	4
**Sigmoid colon**	12
pT stage*	
**T2**	7
**T3**	19
pN stage*	
**N0**	16
**N1**	8
**N2**	2
Advanced tumors**	
**pN+**	10
**M+**	4
Early tumors***	
**pT2 N0**	6
**pT3 N0**	10
**Tumor Type**	
**Mucinous**	0
**Non-mucinous**	30

*Four patients did not undergo resection because of metastatic disease, hence the histopathological T stage (pT) and N stage (pN) were not confirmed and the total in these categories is 26 and NOT 30.

**lymph node metastasis was confirmed pathologically after resection, hence the prefix “p”, the distant metastatic disease in 4 patient was confirmed with imaging in 3 patients and during operative exploration in 1 patient.

***All patients in the early tumor category received resection of primary tumor and regional lymph nodes which were assessed pathologically hence the prefix “p”.

### MR imaging

Imaging was performed with a 1.5T MRI (Ingenia, Philips Medical Systems, Best, The Netherlands) using a phased array body coil. Patients were placed in feet first supine position. Bowel preparation consisted of ≥ 3 hour fasting before the MR examination. To minimize peristaltic movements, patients received an intravenous bolus injection before the MR examination of either 20 mg Hyoscine Butylbromide (Buscopan, Boehringer Ingelheim BV, Ingelheim, Germany) or 1mg of glucagon (GlucaGen Novo Nordisk, Bagsværd, Denmark) reserved for contra-indications to Hyoscine Butylbromide administration.

The protocol consisted of an axial diffusion-weighted sequence using the free breathing method, acquired in 3 stacks; b0, b800 and b1000. The echo time (TE) and repetition time (TR) were 65ms and 3808ms respectively. The slice thickness was 8 mm. The minimal slice gap was 0 mm with a field of view of 380 x 290 mm; the acquisition matrix was 152 x 115 with an acquisition voxel size (mm) of 2.50 x 2.51 x 8.00. The number of excitations (NEX) was 4. Apparent diffusion coefficient maps in gray scale were automatically generated by the operating system using a mono-exponential decay model including all three b-values. For staging purposes, additional T2-weighted turbo spin echo (TSE) sequences using the breath hold technique (2 axial stacks and 1 coronal stack) were acquired. The echo time (TE) and repetition time (TR) were 80 ms and 5596 ms. The slice thickness was 3 mm. The minimal slice gap was 3 mm with a field of view of 390 x 390 mm; the acquisition matrix was 392 x 392 with an acquisition voxel size (mm) of 0.99 x 0.99 x 3.00. The number of excitations (NEX) was 2. The combined acquisition time of these DWI and T2 sequences was 15 minutes.

### Image evaluation and calculation of ADC metrics

Two blinded readers, determined the lymph node stage (N-stage) using the T2 sequences and DWI of all included patients. Both readers are abdominal radiologists with 13 and 8 years of experience in reading abdominal MRI respectively. For positive lymph node metastasis, the criteria were a short axis diameter of ≥8 mm and/or a cluster of 3 or more lymph nodes with a short axis diameter of >5 mm. All patients were classified into two groups by both readers; negative lymph node metastasis (N0) or positive lymph node metastasis (N+). The sensitivity and specificity in predicting lymph node metastasis was calculated for both readers, with histopathology results as the gold standard and the sensitivity and specificity for the prediction of lymph node metastasis were calculated for both readers using a 2x2 contingency table. Subsequently all images were transferred to an offline workstation and analyzed using the IntelliSpace Discovery research platform (Philips Healthcare, The Netherlands). An abdominal radiologist with 13 years experience in reading abdominal MRI and a radiology resident delineated each tumor in consensus directly on the ADC map whilst referring to the b1000 DW images and the T2-weighted sequences for anatomical reference. Tumor boundaries were traced manually on every image containing tumor to include the whole tumor volume in the volume of interest (VOI). Mean ADCs for the whole tumor were calculated from these VOI’s. In addition, the following histogram ADC metrics were calculated: Kurtosis, Skewness and 5^th^, 15^th^, 30^th^, 50^th^, 70^th^, 85^th^, 95^th^ percentiles.

### Standard of reference

Histopathological assessment of the surgical resection specimen served as the primary standard of reference. Patients that did not undergo resection of the primary tumor because of distant metastatic lesions (peritoneum/liver/bone) were diagnosed with obvious appearance on imaging or by direct visualization of metastatic disease (i.e peritoneal implants that were not visible with imaging) during surgery. Patients were classified as advanced colon cancers in case of metastatic disease with at least one of the two following parameters: lymph node metastasis (N+) or distant metastasis (M+).

### Statistical analyses

Statistical analyses were performed with SPSS software version 22.0 (IBM corporation, Armonk, New York, U.S.A). The Mann Whitney U-test was used to compare the ADC and histogram metrics between early and advanced colon cancer tumors. Receiver Operator Characteristics (ROC) analysis was performed to evaluate the diagnostic performance of the histogram ADC metrics to identify advanced tumors. Area under the curve (AUC) and corresponding 95% confidence intervals were calculated. Optimal cutoffs were derived from the ROC-curves (according to the point nearest to the upper left corner) and used to calculate sensitivity, specificity, positive and negative predictive values. *P* -values < 0.05 were considered statistically significant.

## Results

### Patient characteristics

Baseline patient characteristics and following details are reported in Table 1. In total 14 out of 30 (47%) patients had an advanced tumor: Three patients were diagnosed with distant metastasis based on staging imaging (M+); one patient had peritoneal metastasis, one patient had liver metastasis and one patient bone metastasis. One patient was diagnosed with peritoneal metastatic implants through surgical inspection during the initial stage of curative surgery (after which the procedure was cancelled), these peritoneal metastatic implants were not evidently visible on staging imaging. Ten patients had lymph node metastasis with a T2 or T3 tumor (T2/T3 N+), which was confirmed by histopathology after resection. The remaining 16 out of 30 (53%) patients were classified as early tumors (N0 and M0), six had a T2 tumor and the other 10 patients had a T3 tumor, which was confirmed by histopathology after resection. All patients were diagnosed with (histologically proven) non-mucinous type colon adenocarcinoma.

### ADC in advanced versus early tumors

**Fig 1:**
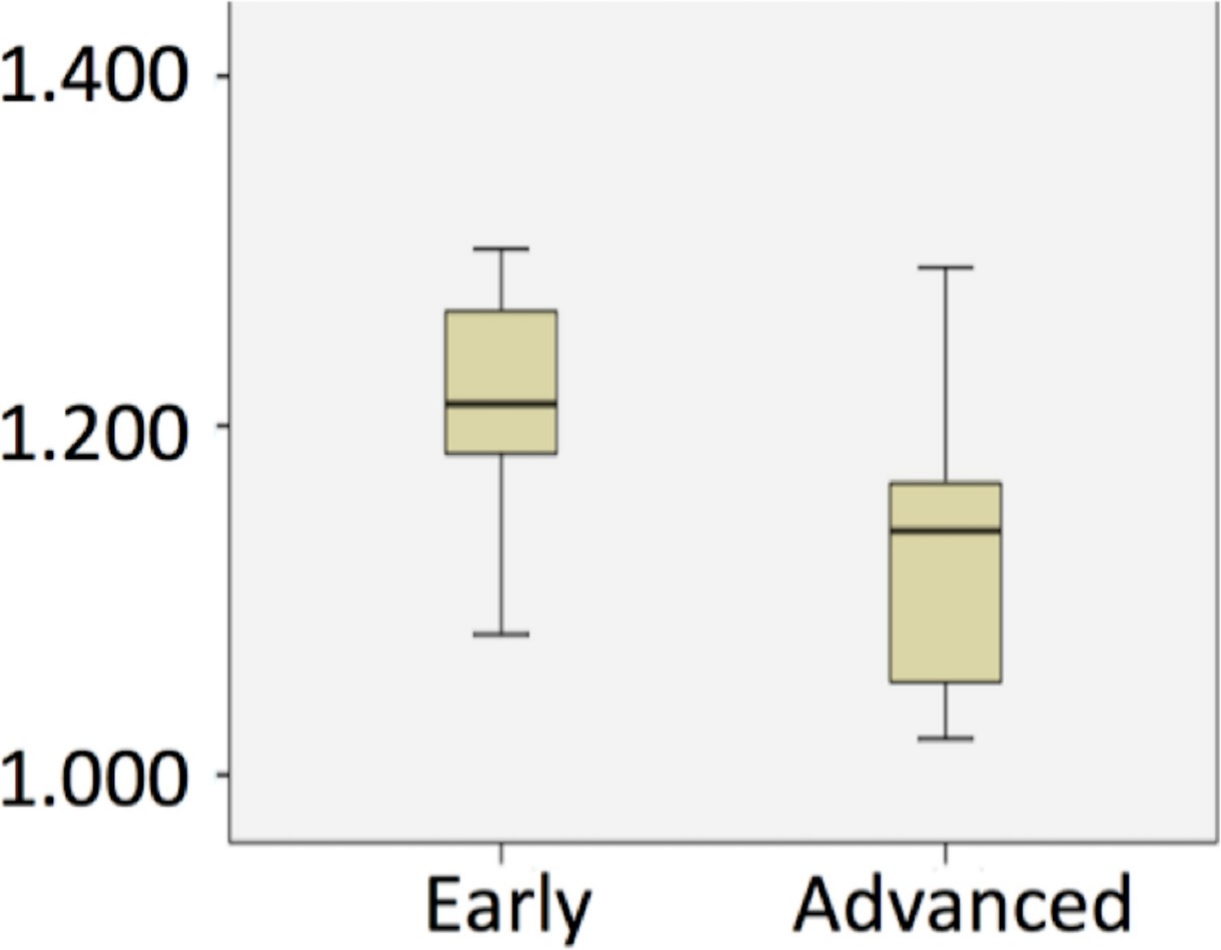
Boxplots for the mean ADC value for early and advanced tumors. ADC values given in mm^2^/s x 10^−3^. ADC = apparent diffusion coefficient.

Table 2 compares the mean ADC and ADC histogram metrics between the advanced versus early tumors. Mean ADC was 1.231±0.118 *10^−3^ mm^2^/s for the early tumors and 1.130±0.072 *10^−3^ mm^2^/s for the advanced tumors (P = 0.002), see boxplot in Fig 1. There were also significant differences in 30^th^, 50^th^ (median) and 70^th^ percentile ADC between the two groups (P = 0.028, P = 0.002 and P = 0.01 respectively).

**Table 2:** Analysis overview of correlation between ADC value and early versus advanced tumor patients. The numbers between the brackets (SD) indicate the standard deviation. ADC = apparent diffusion coefficient; ADC values given in mm^2^/s x 10^−3^.

	Early tumors(SD)	Advanced tumors(SD)	p Value*
**Kurtosis**	4.1 (±2.9)	3.4 (±1.4)	0.98
**Skewness**	0.6 (±0.6)	0.4 (±0.3)	0.58
**ADC**			
**Mean**	1.231 (±0.118)	1.129 (±0.072)	0.002
**5th percentile**	0.832 (±0.147)	0.802 (±0.106)	0.377
**15th percentile**	0.953 (±0.113)	0.907 (±0.062)	0.070
**30th percentile**	1.070 (±0.100)	0.994 (±0.058)	0.028
**50th percentile**	1.206 (±0.120)	1.102 (±0.060)	0.002
**70th percentile**	1.359 (±0.172)	1.236 (±0.090)	0.010
**85th percentile**	1.515 (±0.217)	1.372 (±0.146)	0.064
**95th** **percentile**	1.717 (±0.261)	1520 (±0.219)	0.520

*Mann Whitney Test.

### Diagnostic performance

**Fig 2:**
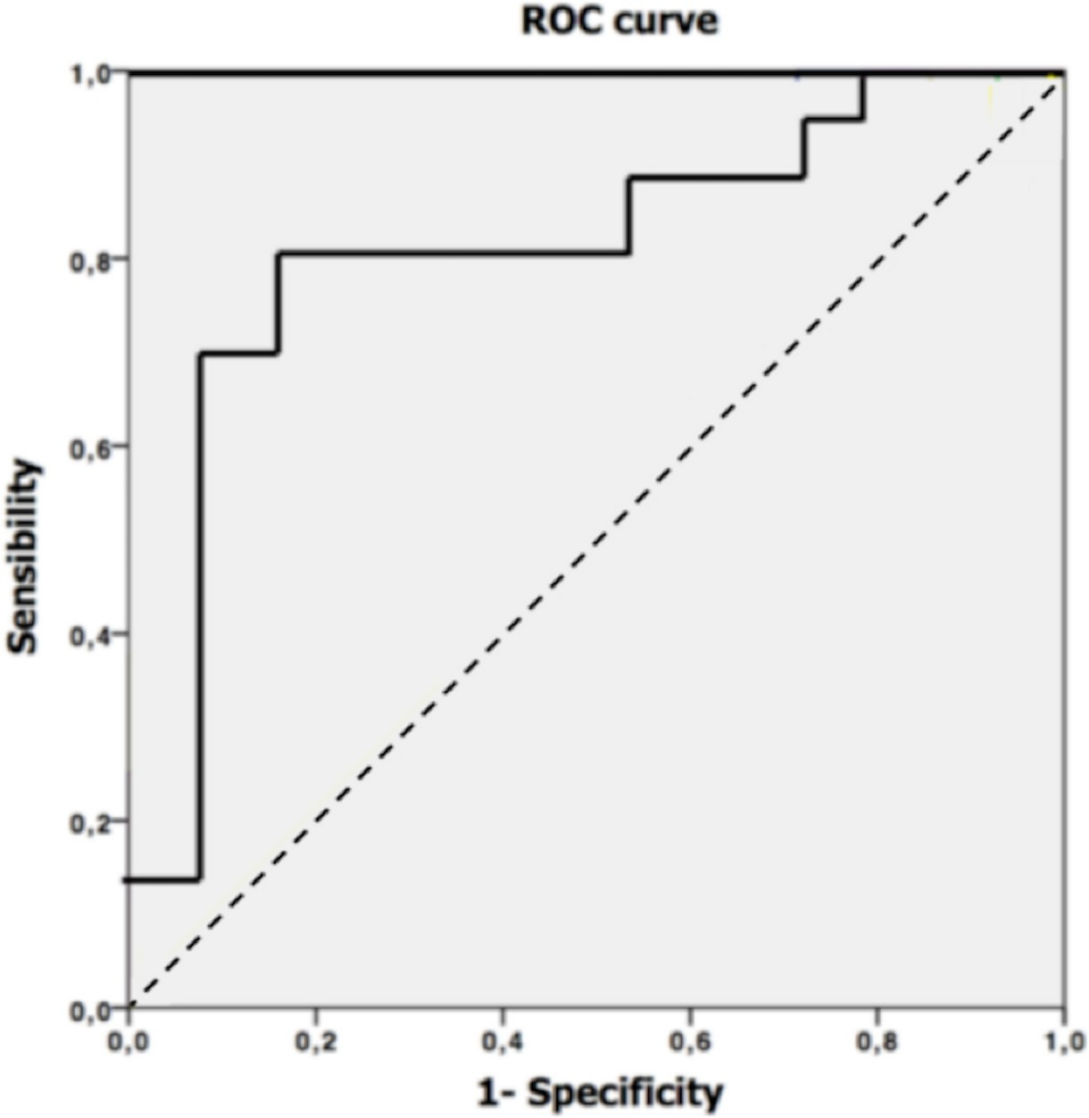
ROC curve for optimal cut of value. Optimal cut off value receiver operating characteristic (ROC) curve of the mean ADC with an area under the curve (AUC) of 0.83.

**Fig 3:**
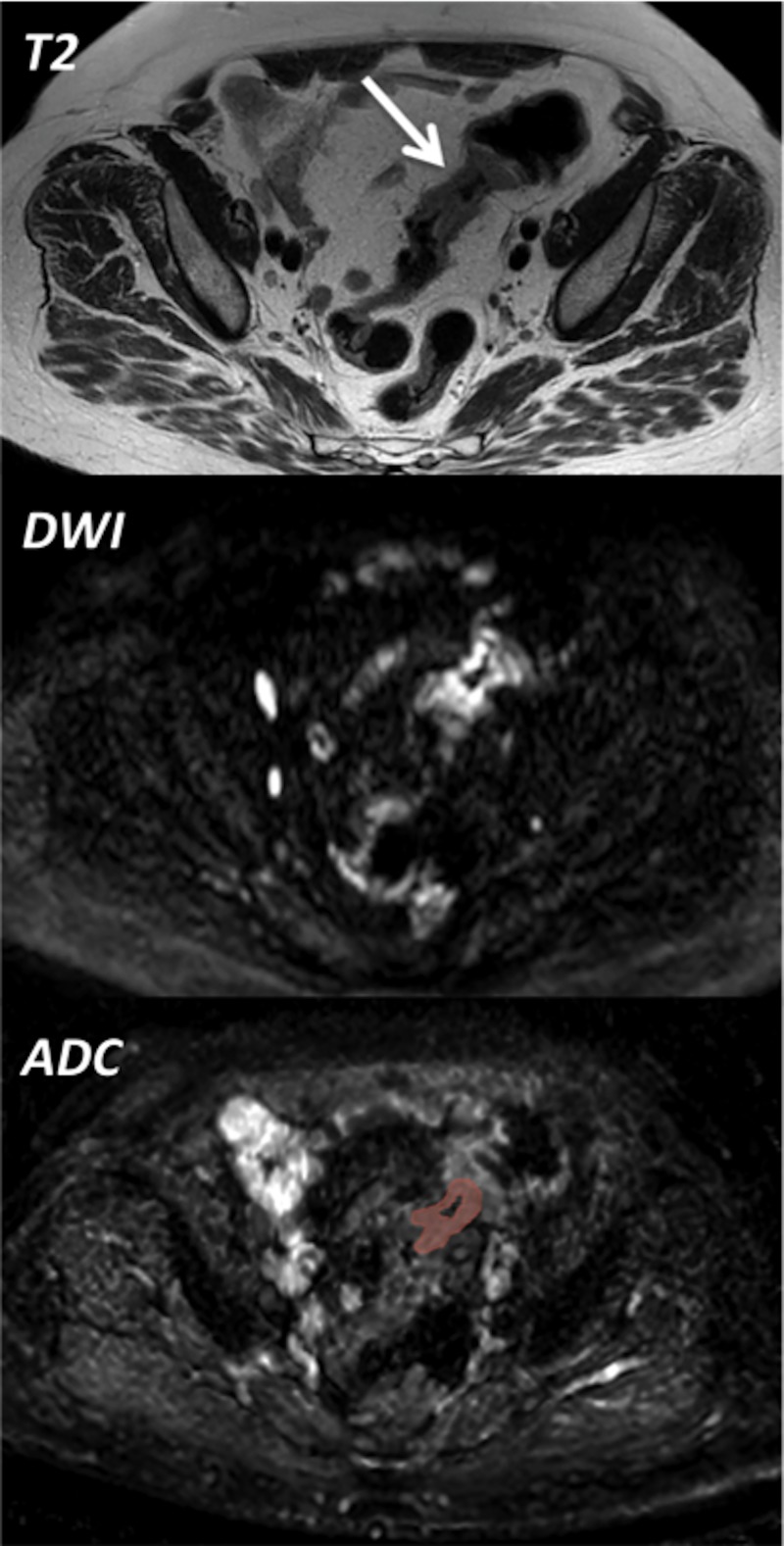
Example of image processing. The T2 weighted MRI sequence (top picture) is showing the tumor in the sigmoid colon (arrow). The middle picture is the corresponding b1000 weighted DWI (Diffusion Weighted Imaging) sequence showing a high signal intensity within the tumor (consistent with malignancy). Bottom picture shows an example of tumor delineation on the corresponding ADC (Apparent Diffusion Coefficient) as performed to obtain the mean ADC-value of the whole tumor. An abdominal radiologist with 13 years experience in reading abdominal MRI and a radiology resident delineated each tumor in consensus directly on the ADC map whilst referring to the T2 weighted images (top) and the DWI sequence (middle) for anatomical reference.

Optimal cut off values, AUC’s, corresponding sensitivities and specificities are provided in Table 3 for the parameters for which a significant difference was found between advanced and early tumors. Best results were obtained for the whole tumor mean ADC using the cut off value of 1.179*10^−3^ mm^2^/s. This cut off value resulted in an AUC of 0.83 (Fig 2) with a sensitivity and specificity of 81% and 86% respectively. In total 25/30 patients were correctly diagnosed (12/14 patients with distant metastasis (M+) or lymph node metastasis (N+) and 13/16 patients without distant (M0) or lymph node metastasis (N0)). An example of a colon tumor (T2, DWI and ADC) is provided in Fig 3.

**Table 3:** Diagnostic performance of ADC to differentiate between early and advanced tumor patients. The numbers between the brackets indicate the p-value. ADC = apparent diffusion coefficient; ADC values given in mm^2^/s x 10^−3^.

	AUC	Cut-off	Sensitivity (%)	Specificity (%)
**Mean**	0.83	1.179	81	86
**30th percentile**	0.74	1.024	75	79
**50th percentile**	0.82	1.154	81	86
**70th percentile**	0.78	1.264	88	64

### Ability of ADC predicting lymph node metastasis (N+)

The accuracy in predicting lymph node metastasis based on morphological criteria by both readers (as compared to histopathological results) are reported in Table 4. The sensitivity and specificity for the prediction of lymph node metastasis were 40% and 63% for reader 1 and 30% and 88% for reader 2 respectively. The whole tumor mean ADC-value using 1.179 * 10^−3^ mm^2^/s as threshold had a 100% sensitivity and specificity in predicting lymph node metastasis in our patient population (Table 4).

**Table 4:** 2x2 contingency tables for the morphological accuracy in detecting lymph node metastasis for reader 1, reader 2 and the accuracy of the apparent diffusion coefficient (ADC) in detecting lymph node metastasis using the threshold value of 1.179 * 10–3 mm2/s.

**Reader 1**	**pN+**	**pN-**
**N+ (MRI)**	4	6
**N- (MRI)**	6	10
**Total**	10	16
*Sensitivity	40% (12.2%-73.8%)	
*Specificity	63% (35.4%-84.8%)	
**Reader 2**	**pN+**	**pN-**
**N+ (MRI)**	3	2
**N- (MRI)**	7	14
**Total**	10	16
*Sensitivity	30% (6.7%-65.3%)	
*Specificity	88% (61.7%-98.5%)	
**ADC**	**pN+**	**pN-**
<1.179 * 10–3 mm2/s	10	0
>1.179 * 10–3 mm2/s	0	16
**Total**	10	16
*Sensitivity	100% (69.2%-100.0%)	
*Specificity	100% (79.4%-100.0%)	

*The numbers behind the brackets represent the 95% confidence intervals.

## Discussion

This study suggests that the ADC value of a colon tumor can be used as a biomarker to predict tumor aggression. Advanced tumors (i.e. tumors with lymph node metastasis/distant metastasis) had significantly lower mean ADC values than early tumors. According to our results the optimal ADC cut off value to discriminate between early and advanced tumors is 1.179 * 10^−3^ mm^2^/s.

It seems tumor aggression correlates with a low ADC value due to the extracellular and cellular architecture of malignant tumors, which impairs the normal movement of water molecules (i.e. Brownian movement).

Another very recent study did investigate the correlation between advanced colon tumors (including N0 vs. N+) and ADC with negative results[24]. This study however included sigmoid tumors only and ADC map was used delineating the most restrictive part of the solid tumor on a single slice (unlike the whole tumor VOI technique used in our study). The authors of this study recognized that the inter-observer agreement of ADC measurements might have been better using whole-volume ROIs, which seem easier reproducible than single slice ROI measurements, as described in a recent rectal cancer study[25].This finding might have a considerable clinical impact as the treatment of colon cancer seems on the brink of a paradigm shift; guidelines for neoadjuvant treatment should become available according to multiple small studies and case reports of patients with advanced tumors [2–6]. If these results are confirmed by the large multicenter setting provided by the FOXTROT-trial[7] imaging must be able to select those patients who could benefit from neoadjuvant therapy. However, a recent meta-analysis [8] showed disappointing accuracy of CT in the prediction of important prognostic factors such as tumor invasion depth of >5 mm in the pericolonic fat the (i.e. T1-3ab versus T3cd-4 tumors); the sensitivity was 77% and specificity was 70%. In addition the sensitivity and specificity for the prediction of lymph node metastasis was 71% and 67%, respectively. This suggests that CT cannot be used as a reliable selection tool to differentiate advanced from early colon tumors.

MRI is a logical alternative given its superior soft tissue contrast and the fact that it already routinely used in staging rectal cancer[9]. Recent studies focusing on colon cancer staging with MRI demonstrated superior results with a sensitivity of 91% and specificity of 84% in discriminating between T1/T2 versus T3/T4 tumors [12] and a sensitivity of 92% and specificity of 94% in discriminating T1-T3ab versus T3cd/T4 tumors [10]. Unfortunately, predicting lymph node metastasis also seems unreliable with MRI [10–12]. This was confirmed by our results provided by the 2 independent readers interpreting the MRI images in our study, with a sensitivity and specificity of 40% and 63% for reader 1 and 30% and 88% for reader 2 respectively. Using ADC as a biomarker, might have been very helpful as all 10 of the patients with N+ disease had an ADC tumor value under 1.179 * 10^−3^ mm^2^/s.

Conversly, the ADC tumor value was higher than 1.179 * 10^−3^ mm^2^/s in all 16 patients that did NOT have lymph node metastasis (or distant metastasis). This entails that the ADC Tumor value has a sensitivity and specificity of 100% in our patient population. Three of the four patients with distant metastasis (M+) were correctly diagnosed with staging imaging, in these cases employment of ADC tumor value would not have had any consequence. One of the four patients with distant metastasis patient however had peritoneal metastasis which was not evidently visible on imaging and became obvious during the initial phase of the curative surgical procedure, when the surgeons noticed multiple peritoneal implants. According to our ADC tumor value results, this patient would have been staged correctly into the advanced tumor group, hence in this case the employment of ADC tumor value would have had a potentially major consequence.

In this regard, additional quantitative MR biomarkers (i.e. ADC) might be considered when selecting patients who could benefit from neoadjuvant chemotherapy. Our results are in line with previous studies in rectal cancer, which found lower ADC values in tumors with a more aggressive profile. In 2012 a study by Curvo-Semedo et al.[14] investigated a potential correlation between ADC values and aggression of rectal cancer tumors. This study demonstrated a significant relationship between a low ADC value and prognostic factors related to a more aggressive tumor profile such as involvement of the mesorectal fascia (MRF+), nodal involvement (N+) and histological differentiation grades. Subsequently, more studies emerged and confirmed the correlation between ADC and tumor aggression in rectal cancer [15–17]. Histologically, there are many similarities between the colon and rectum[26]. This could explain the correlation between tumor aggressiveness and the ADC value in colon cancer as well. It should also be addressed the histogram analyses showed limited benefit with a significant value only for the 30^th^, 50^th^ (median) and 70^th^ percentile. This, again, seems to be similar as in rectal cancer, according to a very recent study in which ADC histogram analyses of rectal tumors were not beneficial to obtain prognostic information [27].

In addition to quantitative MR biomarkers, there are several advantages in the use of MRI in colon cancer staging. First, imaging of the colon can be combined with the imaging of the liver; MRI is the optimal modality in the detection of liver metastasis (for lesions smaller than 10 mm) and the sensitivity of MR imaging is significantly higher than that of CT [28]. Secondly, MRI does not require the use of ionizing radiation and nephrotoxic contrast agents. These promising factors may contribute to a paradigm shift in the diagnostic work-up of colon cancer.

### Limitations

Our study was limited by its retrospective nature and moderate number of inclusions. This was primarily due to the innovative concept of preoperative staging of colon cancer with MRI.

Furthermore our study used two readers in consensus delineating each tumor (instead of 2 independent readers) this may pose a limitation.

However a few studies showed a good interobserver agreement in whole tumor volume analysis using ADC [25] [29] in rectal cancer. Furthermore the whole tumor volume analysis has a better reproducibility than the single slice method[30].

## Conclusion

Our study demonstrates that colon cancer tumors with lymph node metastasis or distant metastasis have significantly lower ADC values than colon tumors without lymph node/distant metastasis. Furthermore, employing tumor ADC values seem more accurate in the prediction of lymph node metastasis instead of relying on the notoriously unreliable morphological characteristics. Consequently, ADC tumor values can help identify patients potentially eligible for neoadjuvant treatment using not only qualitative, but also quantitative biomarkers.
